# The new nitric oxide donor cyclohexane nitrate induces vasorelaxation, hypotension, and antihypertensive effects via NO/cGMP/PKG pathway

**DOI:** 10.3389/fphys.2015.00243

**Published:** 2015-08-31

**Authors:** Leônidas das G. Mendes-Júnior, Driele D. Guimarães, Danilo D. A. Gadelha, Thiago F. Diniz, Maria C. R. Brandão, Petrônio F. Athayde-Filho, Virginia S. Lemos, Maria do S. França-Silva, Valdir A. Braga

**Affiliations:** ^1^Biotechnology Center, Federal University of ParaíbaJoão Pessoa, Brazil; ^2^Department of Physiology and Biophysics, Institute of Biological Sciences, Federal University of Minas GeraisBelo Horizonte, Brazil; ^3^Department of Chemistry, Federal University of ParaíbaJoão Pessoa, Brazil

**Keywords:** nitric oxide, nitric oxide donors, mesenteric artery, KATP channels, hypertension

## Abstract

We investigated the cardiovascular effects induced by the nitric oxide donor Cyclohexane Nitrate (HEX). Vasodilatation, NO release and the effects of acute or sub-chronic treatment with HEX on cardiovascular parameters were evaluated. HEX induced endothelium-independent vasodilatation (Maximum effect [efficacy, ME] = 100.4 ± 4.1%; potency [pD2] = 5.1 ± 0.1). Relaxation was attenuated by scavenging nitric oxide (ME = 44.9 ± 9.4% vs. 100.4 ± 4.1%) or by inhibiting the soluble guanylyl cyclase (ME = 38.5 ± 9.7% vs. 100.4 ± 4.1%). In addition, pD2 was decreased after non-selective blockade of K^+^ channels (pD2 = 3.6 ± 0.1 vs. 5.1 ± 0.1) or by inhibiting K_ATP_ channels (pD2 = 4.3 ± 0.1 vs. 5.1 ± 0.1). HEX increased NO levels in mesenteric arteries (33.2 ± 2.3 vs. 10.7 ± 0.2 au, *p* < 0.0001). Intravenous acute administration of HEX (1–20 mg/kg) induced hypotension and bradycardia in normotensive and hypertensive rats. Furthermore, starting at 6 weeks after the induction of 2K1C hypertension, oral treatment with the HEX (10 mg/Kg/day) for 7 days reduced blood pressure in hypertensive animals (134 ± 6 vs. 170 ± 4 mmHg, respectively). Our data demonstrate that HEX is a NO donor able to produce vasodilatation via NO/cGMP/PKG pathway and activation of the ATP-sensitive K^+^ channels. Furthermore, HEX acutely reduces blood pressure and heart rate as well as produces antihypertensive effect in renovascular hypertensive rats.

## Introduction

Arterial hypertension is a chronic condition characterized by high levels of blood pressure (BP) and is considered the main cause of morbidity and mortality worldwide (Chobanian, 2011; World Health Organization, 2013). The interaction between numerous factors such as genetic, physiological, and environmental characteristics has been implicated in the pathophysiology of hypertension (Montezano and Touyz, 2012). Currently, it has been well established that the decreased in nitric oxide (NO) levels and reduced antioxidant capacity in the vessels, heart, brain, and kidneys may play a key role in the pathogenesis and maintenance of arterial hypertension (Puddu et al., 2008; Sugamura and Keaney, 2011).

Nitric oxide (NO) is the major endothelium-derived relaxing factor (EDRF) produced when nitric oxide synthase (NOS) family catabolizes the conversion of amino acid l-arginine to l-citrulline (Palmer et al., 1988). NO participates in maintaining and regulating BP due its role in the adjustments of systemic vascular resistance (Wilkinson et al., 2004). In vascular smooth muscle cells (VSMCs), NO activates the soluble guanylyl cyclase (sGC) enzyme producing a second messenger called cyclic guanosine monophosphate (cGMP) that activates the specific cGMP-dependent protein kinase (PKG), which is the main kinase mediating vasodilation (Walford and Loscalzo, 2003; Bryan et al., 2009). In addition, NO can directly activate protein and ion channels to promote vasodilation (Bolotina et al., 1994). An enhanced inactivation and/or reduced synthesis of NO are related to cardiovascular disorders such as hypertension (Massion et al., 2005; Naseem, 2005).

**Figure 1:**
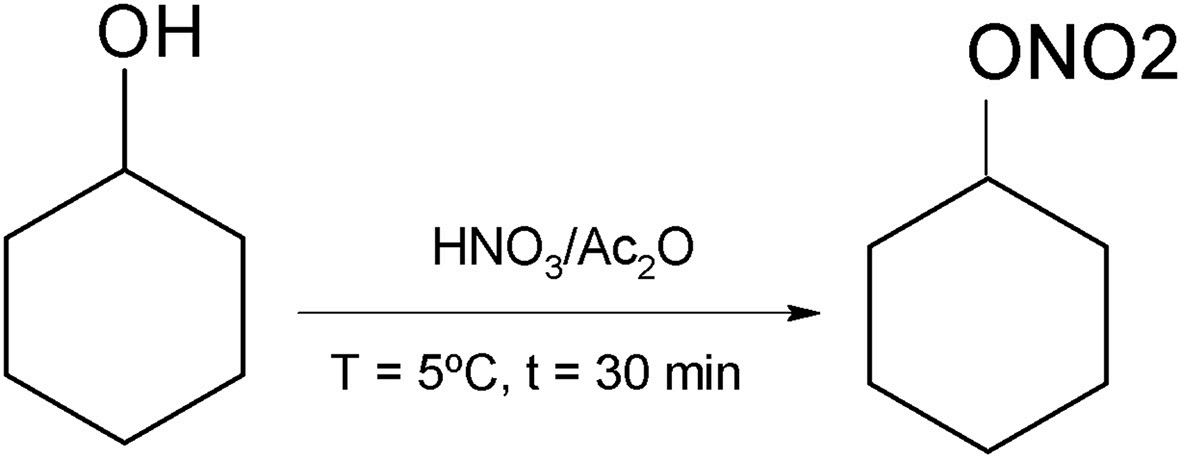
**Synthetic route for obtaining the cyclohexane nitrate (HEX)**.

Therapies targeting to increase NO levels have great applicability in cardiovascular diseases. NO donors (e.g., nitroglycerin and sodium nitroprusside) have been used in clinical practice for decades against angina pectoris, pulmonary hypertension, and cardiac ischemia (Münzel et al., 2011). However, tolerance developed after the long-term use of these agents impairs the chronic treatment of hypertension (Napoli and Ignarro, 2003; Csont and Ferdinandy, 2005). Considering the clinical potential for NO donors, we synthesized the cyclohexane nitrate (HEX) from cliclohexanol as illustrated in the synthetic route presented in Figure 1. Therefore, our aims were: (a) characterize this novel organic nitrate as a NO donor; and (b) evaluate its effects on cardiovascular system of normotensive and hypertensive rats. Our data show that HEX increases NO levels in endothelium-denudated mesenteric arteries, promoting vasorelaxation by the activation of NO/cGMP/PKG pathway and ATP-sensitive K+ channel (K_ATP_). Furthermore, we experimentally demonstrated that HEX is able to reduce blood pressure in hypertensive rats.

## Materials and methods

### Animals

All procedures were approved by Federal University of Paraiba Animal Care and Use Committee (CEUA/UFPB # 604/14). Male Wistar rats (250–300 g) were housed in a temperature-controlled room, 12:12-h light–dark cycle with free access to standard rat chow (LabinaⓇ, Purina, Paulinea, SP, Brazil) and water. Renovascular hypertension (2K1C model) was induced in rats as previously described (Botelho-Ono et al., 2011; Mendes-Junior et al., 2013). In brief, under combined ketamine and xylazine anesthesia (75 and 10 mg/kg, i.p., respectively), a midline abdominal incision was made. The right renal artery was exposed and isolated over a short segment by blunt dissection. A U-shaped silver clip (0.2 mm internal diameter) was placed over the vessel at a site proximal to the abdominal aorta and the wound closed and sutured. A sham procedure, which entailed the entire surgery with the exception of renal artery clipping, served as control. After the procedures, rats returned to their home cages and remained untouched for 6 weeks in order to develop hypertension.

### Synthesis of cyclohexane nitrate

In volumetric balloon (100 mL) equipped with a magnetic agitator and a thermometer, 10 mL of acetic anhydride (0.06 mol) was added, followed by 10 mL of fuming nitric acid (0.06 mol). The temperature was set to 5°C. The amount of fuming nitric acid required was slowly added to the solution. Next, 10 mL of ciclohexanol (0.05 mol) was added to the solution. The balloon was agitated at 200 rpm for 30 min. The reaction was stopped by adding cold distilled water (2x the initial volume of the solution). The reaction results in a biphasic solution. The aqueous phase is neutralized by adding sodium bicarbonate and the organic phase is treated with sodium sulfate to remove humidity. The reaction is illustrated in Figure 1.

### Nitric oxide measurements by epifluorescence microscopy

Normotensive animals were euthanized and the cranial mesenteric artery was collected and sectioned in rings (2–3 mm). The rings were kept in physiological Tyrode's solution (composition in mmol/L: NaCl 158.3; KCl 4.0; CaCl_2_ 2.0; MgCl_2_ 1.05; NaH_2_PO_4_ 0.42; NaHCO_3_ 10.0 and glucose 5.6), at 37°C and pre-incubated for 30 min in the darkness with 4,5-diaminofluorescein diacetate (DAF-2 DA; 10 mM; Calbiochem, San Diego, CA, USA), an intracellular fluorescent probe for NO. Tissues were washed and incubated with: HEX (1 mM); HEX (1 mM) + L-NNA (100 μM); GTN (10 μM); or GTN (10 μM) + L-NNA (100 μM) for 30 min. A series of experiments was performed only by incubating the probe to measure the baseline fluorescence. For cryostat sectioning, tissues were previously fixed in OCT embedding compound and frozen at –20°C. Sections (8 μm) were cut on a CM 1900 cryostat (Leica Inc., Deerfield, IL, USA) and mounted on slides and cover slips. The 4′,6-diamidino-2-phenylindole stain (DAPI) was used to assist with the cell nucleus observation. The cytosolic NO level was assessed by exciting DAF-2 DA in 480 nm using a xenon lamp and measuring the fluorescence at 490–530 nm in epifluorescence microscope (Nikon Eclipse TI, Tokyo, Japan). The relative fluorescence intensity was calculated from images obtained using a digital camera (DEI-470, Optronics Engineering, Goleta, CA) and ImageJ software 1.42q (Wayne Rasband, National Institutes of Health, USA). The NO content was obtained by the change in the intensity of fluorescence (IF), which was calculated by the difference between the final (IFf) and initial (IFi) florescence converted in percentage of the baseline as %ΔIF = (IFf – IFi/IFf) × 100.

### Vascular reactivity studies

Cranial mesenteric artery rings (1–2 mm) were obtained as described above and mounted vertically on two Δ-shaped stainless steel wires in a 10-ml tissue chamber connected to a tension transducer (PowerLab™, ADInstruments, MA, EUA). The preparations were stabilized under 0.75 g resting tension for 1 h. The tissue viability was verified by phenylephrine (PHE, 10 μM) added to the bath and the presence of functional endothelium was assessed by acetylcholine (ACH, 10 μM). Experiments have been conducted in intact and endothelium-denudated mesenteric artery rings. Mesenteric artery rings were pre-contracted using PHE (1 μM). After the contraction plateau was reached, HEX (10^−10^–10^−3^ mM) was added to the organ bath in order to build a concentration-response curve. Data were expressed as maximum effect (ME) and potency (pD2). The ME is related to the efficacy of the drug while the pD2 refers to the potency of a drug. pD2 is the negative logarithm to base 10 of the EC_50_. In independent protocols, the preparations were pre-incubated (30 min) with: 2-phenyl-4,4,5,5-tetramethylimidazoline-1-oxyl 3-oxide (PTIO, 300 μM), a NO scavenger or 1H-[1,2,4]oxadiazolo[4,3-a]quinoxalin-1-one (ODQ, 10 mM), a soluble guanylyl cyclase inhibitor or tetraethylammonium (TEA, 3mM), a non-selective K^+^ channels blocker or TEA, 1 mM, a large conductance Ca^2+−^sensitive K^+^ channel blocker or glibenclamide (GLIB, 1 mM), a K_ATP_ blocker or 4-aminopyridine (4-AP, 1 mM), a voltage-operated K^+^ channel blocker. All drugs were obtained from Sigma-Aldrich (São Paulo-SP, Brazil). ODQ and GLIB were solubilized in DMSO and the others compounds were dissolved in distilled water. In functional studies using ODQ and GLIB, the final concentration of DMSO never exceeded 0.01% in the bath and had no effect when tested in control preparations (data not shown).

### Acute changes in cardiovascular parameters of rats treated with HEX

The rats were anesthetized with ketamine and xylazine (75 and 10 mg/kg, intraperitoneal [IP], respectively). Polyethylene cannulae were inserted into the abdominal aorta and inferior vena cava through femoral artery and vein for arterial pressure recordings and drug injections, respectively. Blood pressure and heart rate measurements were recorded 24 h after catheter implantation in conscious rats using a pressure transducer coupled to an acquisition system (PowerLab; ADInstruments, Castle Hill, NSW, Australia) connected to a computer running LabChart 5.0 software (ADInstruments). HEX was dissolved in saline solution and administered through venous catheter after 20 min of baseline recordings. Blood pressure and heart rate were evaluated before and after the administration of HEX (1; 5; 10; 20 mg/kg, intravenous injection [IV], randomly). The interval between doses was 15 min. Data were expressed as changes in mean arterial pressure (ΔMAP) and changes in heart rate (ΔHR).

### Sub-chronic treatment with HEX in hypertensive rats

Animals were anaesthetized with ketamine and xylazine (75 and 10 mg/kg, IP, respectively) and surgically prepared according to AAALAC-recommended aseptic techniques. Telemetric devices (model TRM54P, Telemetry Research, Millar Instruments, United States of America) were implanted according to the procedure described by Burmeister et al. (2011). The animals were divided in three groups: (I) 2K1C treated with HEX in drinking water (10 mg/kg/day) from day 42 to day 49 after renal artery clipping; (II) 2K1C treated with saline and (III) Sham group. The dose of HEX (10 mg/kg/day) has been chosen based on our acute studies, where it produced submaximal effects on blood pressure and heart rate. Blood pressure was recorded continuously for 1 h from 8:00 a.m. to 9:00 a.m., 1 day per week for 7 weeks. Heart rate data were derived from the blood pressure signal offline.

### Statistical analysis

Data are expressed as the mean ± SEM. Statistical analysis was performed by Student's *t*-test or ANOVA Two-Way when appropriate, using the Graph Pad Prism 5.0 program. Differences were considered significant when *p* < 0.05.

## Results

### HEX increases cytosolic NO levels in cranial mesenteric arteries independent on eNOS enzyme

**Figure 2:**
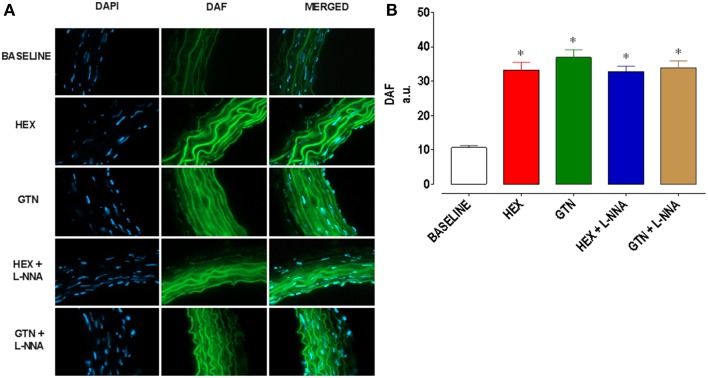
**(A)** Representative images of fluorescence in cranial mesenteric artery sections incubated with DAF-2 DA (10 μmol/L), a fluorescent cell permeable probe for NO. **(B)** Bars graph showing the NO production after incubation with only DAF-2 DA (Baseline) and treatments groups: HEX; nitroglycerin (GTN); HEX + L-NNA; GTN + L-NNA. Fluorescence was quantified by optic densitometry (arbitrary units, a.u.). Results are mean ± s.e.m., *n* = 5 each group. ^*^p < 0.05 vs. Baseline.

Original images from epifluorescence microscopy (Figure 2A) show cranial mesenteric artery sections incubated with DAF-2 DA and the treatments performed. The data plotted on graph (Figure 2B) show at first the baseline fluorescence emitted by the probe without any further treatment (control). After incubation with HEX the fluorescence significantly increased in samples when compared to control (33.23 ± 2.29 vs. 10.66 ± 0.62 a.u., respectively, *p* < 0.05). The nitroglycerin (GTN), the positive control, also increased the fluorescence (36.92 ± 2.25 vs. 10.66 ± 0.62 a.u., respectively, *p* < 0.05). Furthermore, L-NNA did not prevent the increase in fluorescence promoted by both HEX (32.77 ± 1.65 vs. 33.23 ± 2.29 a.u., respectively, *p* > 0.05) and GTN (33.95 ± 1.96 vs. 36.92 ± 2.25 a.u., respectively, *p* > 0.05).

### HEX induces vasorelaxant effect in cranial mesenteric artery of normotensive rats, which is independent on functional endothelium

**Figure 3:**
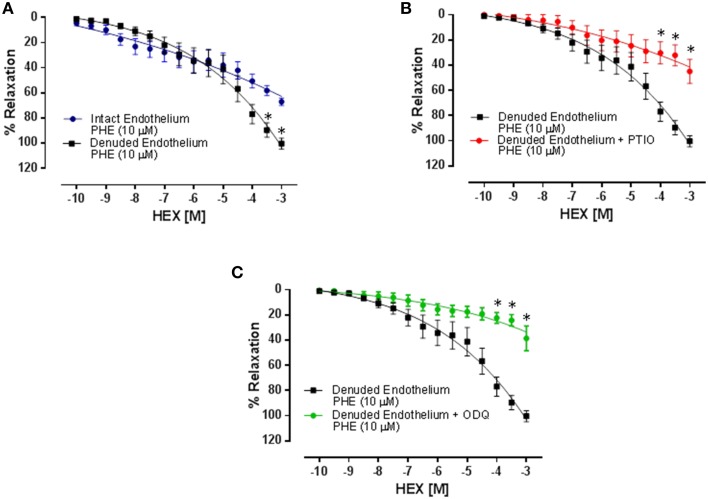
**Relaxation induced by cumulative addition of HEX (10^−10^–10^−3^ M) in cranial mesenteric artery rings pre-contracted with PHE (10 μM)**. Results are mean ± s.e.m. **(A)** Intact Endothelium vs. Denuded Endothelium, ^*^p < 0.05 vs. intact endothelium. **(B)** Denuded Endothelium vs. Denuded Endothelium + PTIO, ^*^p < 0.05 vs. Denuded Endothelium. **(C)** Denuded Endothelium vs. Denuded Endothelium + ODQ, ^*^p < 0.05 vs. Denuded Endothelium.

As shown in Figure 3A, in phenylephrine pre-contracted cranial mesenteric artery rings isolated from normotensive rats, HEX administration (10^−10^–10^−3^ M) produces vasorelaxation in the presence of functional endothelium (ME = 67.0 ± 2.8%; pD2 = 5.84 ± 0.22, *n* = 7). Moreover, after the mechanical removal of the endothelium, the vasorelaxation was potentiated (ME = 100.4 ± 4.1%; pD2 = 5.11 ± 0.12, *n* = 10, *p* < 0.05).

### Vasorelaxation induced by HEX is mediated by nitric oxide release

In endothelium denuded mesenteric artery rings pre-contracted with PHE and in the presence of the NO scavenger PTIO, the vasorelaxant effect of HEX was reduced when compared to the control (ME = 44.9 ± 9.4 vs. 100.4 ± 4.1%, respectively, *n* = 5, *p* < 0.05). Moreover, the sCG inhibitor ODQ caused a great decrease in the vasorelaxant effect induced by HEX, in which the maximum effect was almost abolished when compared to the control (ME = 38.56 ± 9.7% vs. 100.4 ± 4.1%, respectively, *n* = 5, *p* < 0.05), as illustrated in Figures 3B,C.

### Vasorelaxation induced by HEX is mediated by K+ATP channels

**Figure 4:**
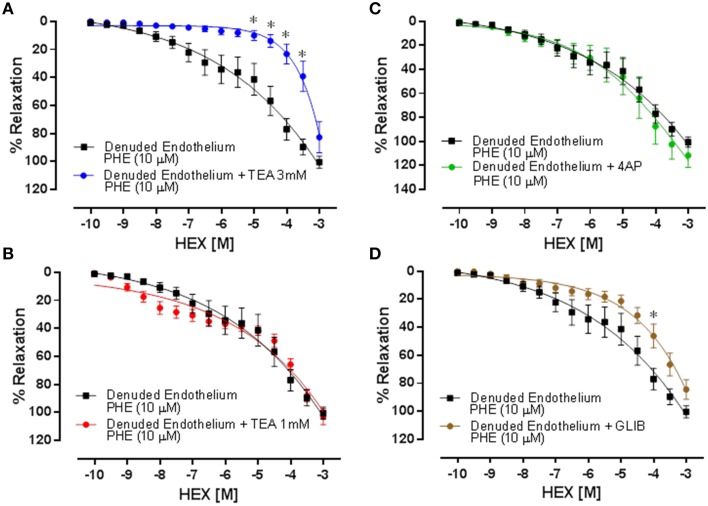
**Relaxation induced by cumulative addition of HEX (10^−10^–10^−3^ M) in cranial mesenteric artery rings pre-contracted with PHE (10 μM)**. **(A)** Denuded Endothelium vs. Denuded Endothelium + TEA (3 mM). **(B)** Denuded Endothelium vs. Denuded Endothelium + TEA (1 mM). **(C)** Denuded Endothelium vs. Denuded Endothelium + 4-AP. **(D)** Denuded Endothelium vs. Denuded Endothelium + GLIB. Results are mean ± s.e.m. ^*^p < 0.05 vs. Denuded Endothelium.

In cranial mesenteric artery rings without functional endothelium, pretreatment with TEA (3 mM), which in that concentration acts as a non-selective K+ channels blocker, shifted the concentration-response curve to the right decreasing the HEX potency (Figure 4A), as observed by the pD2 value when compared with the control (pD2 = 3.65 ± 0.17 vs. 5.11 ± 0.12, respectively, *n* = 5, *p* < 0.05). In order to assess the subtypes of K+ channels, more specific K+ channels blockers were used. The vasorelaxant responses induced by increasing concentrations of HEX were not significantly modified by tissue pre-exposure to TEA (BKCa blocker) or 4-AP (KV blocker). On the other hand, when GLIB (K_ATP_ channel blocker) was used, the HEX-induced vasodilatation was attenuated (pD2 = 4.38 ± 0.09 vs. 5.11 ± 0.12, *n* = 6, *p* < 0.05) without changing in maximum effect as illustrated in Figures 4B–D.

### Acute administration of HEX reduces blood pressure and heart rate in hypertensive rats

**Figure 5:**
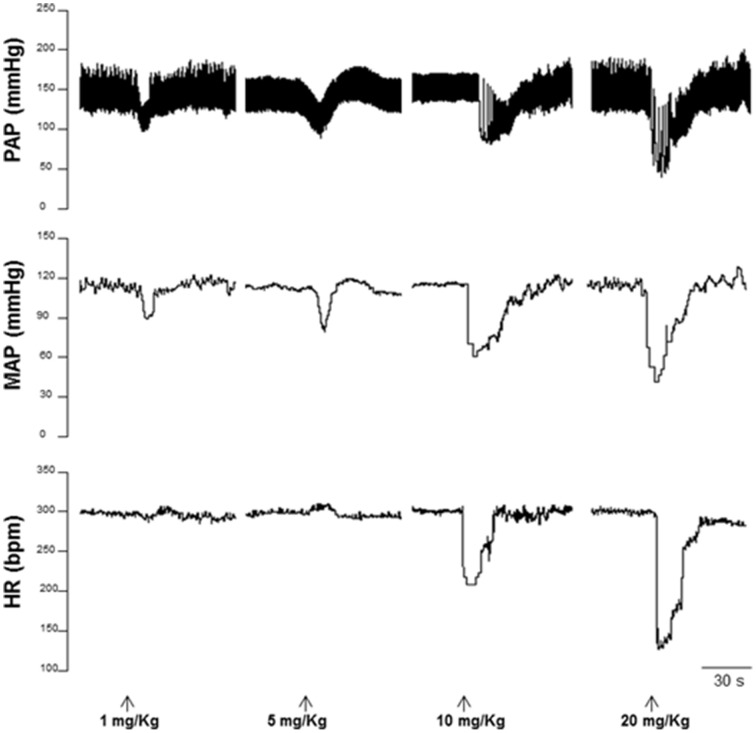
**Representative tracings from a sham operated rat illustrating changes in pulse arterial pressure (PAP, mmHg), mean arterial pressure (MAP, mmHg), and heart rate (HR, bpm) in response to administration of HEX (1; 5; 10; and 20 mg/kg)**.

**Figure 6:**
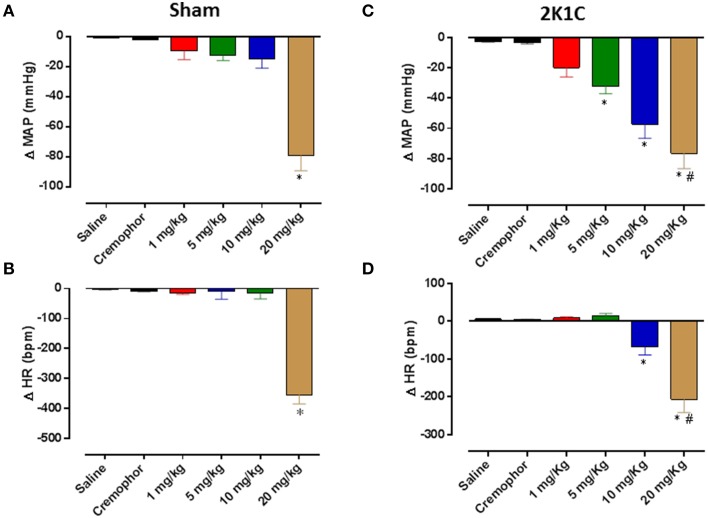
**Hypotensive effect induced by intravenous injections of HEX (1; 5; 10; and 20 mg/kg)**. Changes in mean arterial pressure (ΔMAP) in **(A)** Sham and **(C)** 2K1C rats. Changes in heart rate (ΔHR) in **(B)** sham and **(D)** 2K1C rats. Data presented as mean ± s.e.m. ^*^p < 0.05 vs. saline, ^#^*p* < 0.05 vs. 10 mg/Kg.

Six weeks after 2K1C surgery, the baseline MAP was increased compared with sham-operated rats (145 ± 3 vs. 120 ± 2 mmHg, respectively, *n* = 6, *p* < 0.05). A representative tracing from a sham operated rat is shown in Figure 5. In normotensive rats, HEX (1; 5; 10; and 20 mg/kg) reduced blood pressure (−20 ± 6; −32 ± 5; −57 ± 9; −76 ± 9 mmHg) followed by a slight tachycardia in first two doses (8 ± 2 and 14 ± 6 bpm) and significant bradycardia in last two doses (−68 ± 20 and −206±34 bpm) as shown in Figures 6A,B. Furthermore, in conscious hypertensive rats, HEX (1; 5; 10; and 20 mg/kg) reduced blood pressure in a dose-dependent fashion (−9 ± 5; −12 ± 3; −15 ± 6; −79 ± 10 mmHg, respectively), also followed by bradycardia (−15 ± 3; −8 ± 26; −15 ± 18 and −355±29 bpm) as shown in Figures 6C,D.

### Sub-chronic administration of HEX (v.o.) produces anti-hypertensive effect in renovascular hypertensive rats

**Figure 7:**
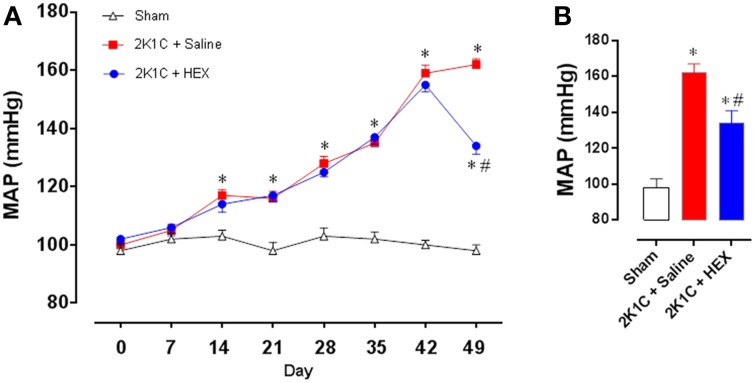
**Effect of sub-chronic administration of HEX (10 mg/kg/day) in Sham, 2K1C + Saline and 2K1C + HEX groups**. **(A)** Time course of the variation in MAP. **(B)** Bar graph showing the MAP in the end of treatments (day 49). Data reported as mean ± s.e.m. ^*^p < 0.05 vs. Sham, ^#^*p* < 0.05 vs. 2K1C + Saline.

BP and HR were also evaluated for 7 weeks using telemetry. As illustrated in Figure 7A, in both 2K1C groups the MAP increased by 3rd week post clipping (118 ± 4 and 117 ± 4 mmHg vs. 110 ± 5 mmHg, *n* = 6 for each group), remaining in ascendancy until the 6th week (157 ± 5 and 154 ± 5 mmHg vs. 106 ± 6 mmHg, *n* = 6 for each group). In the 7th week the 2K1C group treated with vehicle remained hypertensive (170 ± 4 mmHg) while the HEX-treated 2K1C rats showed a significant reduction in MAP (134 ± 6 mmHg vs. 170 ± 4 mmHg, *p* < 0.05) as illustrated in Figure 7B. Sham animals showed no alterations in MAP during the 7 weeks of treatment. In addition, there was no change in heart rate among groups over the 7 weeks of the study (data not show).

## Discussion

The major findings of this study are as follows: (i) HEX is a potential organic nitrate able to release NO and promote relaxation through the activation of the sGC/GMPc/PKG pathway and K_ATP_ channels; (ii) HEX promotes hypotension in normotensive and hypertensive animals; (iii) oral administration of HEX for 7 days induces antihypertensive effect in renovascular hypertensive animals.

The organic nitrates are the most commonly used NO donor drugs and have been used in the treatment of cardiovascular diseases for many years. They are often used in association with beta-blockers drugs and calcium-channel antagonists in the therapy protocol of ischemic and hypertensive patients. The major limitation of organic nitrates used in clinical practice is the rapid development of tolerance following chronic administration (Miller and Megson, 2007). Thus, despite the existence of medicines in the same therapeutic class, it is still necessary to search for new NO donors with clinical utility but fewer side effects.

Nitric oxide donors induce vascular relaxation by NO generation like others nitrates (França-Silva et al., 2012a; Araújo et al., 2013). In this study (Figure 3) we showed that HEX induces endothelium-independent vasodilation in cranial mesenteric artery rings isolated from normotensive rats. In fact, the vasorelaxation had the maximum effect potentiated in rings without endothelium. It means that the vascular endothelium negatively modulates vasorelaxation induced by the nitrate. Bonaventura et al. (2009) showed a similar result, where the concentration-response curve to [Ru(terpy)(bdq)NO](3+)-TERPY, a different NO donor, was less potent in aortic rings with endothelium preserved due to the uncoupling of eNOS, resulting in oxidation of BH4, an important cofactor in the NO production process by eNOS. It is important to highlight that HEX does not cause uncoupling of eNOS, based on our preliminary data showing that HEX does not increase superoxide levels in mesenteric artery (data not shown). Alternatively, secondary products from the metabolism of HEX could induce signaling mechanisms on the endothelial cells attenuating normal vasorelaxation induced by the compound. Of note, both NO scavenger carboxy-PTIO and the sGC inhibitor ODQ diminished the HEX effect confirming the involvement of both NO and sGC in the vasorelaxant effect induced by HEX.

In order to confirm the hypothesis that HEX releases NO, we used the specific probe DAF-2 DA on vascular smooth muscle cells (VSMCs) of cranial mesenteric artery from normotensive rats. Under these experimental conditions, HEX induced an increase in intracellular NO in myocytes analyzed by fluorescence microscopy (Figure 2) indicating that the organic nitrate is metabolized in biological systems releasing NO. Although this technique is extremely accurate as the location of the NO molecule *per se*, the metabolic pathway and the site of generation cannot be predicted. Considering the vascular endothelium as the main physiological source of NO and the absence of studies about the effects of HEX in endothelial cells, especially its actions on the eNOS, we used the eNOS selective inhibitor (L-NNA) to investigate the possible participation of this enzyme in the HEX-mediated increases in NO. Our data show that even after blockade of eNOS with L-NNA, HEX was able to increase the NO levels, indicating that the enzyme is not participating in the effects induced by HEX. Therefore, there is no activation of eNOS with subsequent formation of NO. The results obtained by epifluorescence microscopy confirm the hypothesis that HEX is an organic nitrate able to donate nitric oxide in VSMCs without the involvement of eNOS. It is important to highlight that epifluorescence microscopy for measuring nitric oxide levels has some limitations. In the presence of NO, divalent cations like Ca^2+^ increase DAF-2 fluorescence. The Ca^2+^ sensitivity of DAF-2, thus, makes it difficult to distinguish an intracellular Ca^2+^ increase from an increase in intracellular NO. Measurement of extracellular NO released from cells by DAF-2 should be less prone to this problem and thus might be an alternative to the intracellular bio-imaging by DAF-2 DA. However, this limitation has been questioned by Leikert et al. (2001).

It is well described that nitric oxide binds to guanylyl cyclase, increasing cGMP levels and activating PKG. One of the major target for PKG is the family of potassium channels expressed in VSMCs. Four types of these channels are found in the VSMCs: voltage-activated –K_v_–; BK_Ca_; inward rectifiers –K_ir_– (that include the ATP-sensitive–KATP–) and two-pore domain K^+^ (K2P) channels (Nelson and Quayle, 1995; Cox and Rusch, 2002; Jackson, 2005). Activated K^+^ channels in VSMCs membrane increase K^+^ efflux causing membrane hyperpolarization and consequently decreased in Ca^2+^ entry through voltage-operated Ca^2+^ channels making these channels the main contributor to resting membrane potential and therefore an important determinant of vascular tone (Billaud et al., 2014).

In order to evaluate the activation of potassium channels induced by HEX, we used TEA (3 mM) for unselectively blockade of those K^+^ channels described. Under these experimental conditions, a decrease in nitrate potency was observed, clearly demonstrating that some of those channels might be involved in the vasorelaxant effect induced by HEX, probably via direct activation by NO and indirect activation by the NO/cGMP/PKG pathway. Nevertheless, this experimental protocol cannot specify which channel subtypes were participating in the vasorelaxant effect induced by HEX. Therefore, specific blockers were used for the each subtype of channels expressed in VSMCs. TEA (1 mM) acting as a BK_*Ca*_ blocker and 4-AP (1 mM) as K_v_ blocker did not affect the effects induced by HEX. On the other hand, GLIB (1 mM) shifted the concentration-response curve to the right, indicating the participation of ATP-sensitive K+ channel on the vasorelaxation induced by HEX.

In order to evaluate the physiological significance of HEX, the compound was administered in normotensive and hypertensive rats. As expected, HEX induced reduction in blood pressure in all animals in a dose dependent fashion. The renovascular hypertension model used in this study (2K1C) is recognized as it involves an increase in renin-angiotensin system activity and consequently activation of AT1 receptors (Červenka et al., 2008). Moreover, the 2K1C model is characterized by endothelial dysfunction, vascular remodeling, increased sympathetic activity, all of those via AT_1_R and ROS generation in the CNS and periphery (Intengan and Schiffrin, 2001; Oliveira-Sales et al., 2008; Costa et al., 2009). Emerging evidence suggests that the oxidative stress and endothelial dysfunction have a causal role in the molecular processes leading to hypertension. Reactive oxygen species (ROS) may directly alter vascular function or cause changes in vascular tone by several mechanisms including altered NO bioavailability or signaling (Schulz et al., 2011). Thus, the less potent effect of HEX in hypertensive animals may be due to large concentration of ROS and its interaction with NO.

HEX administration (i.v.) produced bradycardia, unlike other NO donors such as sodium nitroprusside and nitroglycerin (Needleman, 1976; Caputi et al., 1980), which induced tachycardia as a reflex response to the fall in blood pressure. There are evidence showing that NO donors can enhance cardiac vagal neurotransmission by sGC activation, increased acetylcholine release and consequent decrease heart rate (Paton et al., 2002). In addition, other organic nitrate studied in our laboratory also produces bradycardia by direct vagal stimulation dependent of an action in the CNS (França-Silva et al., 2012b). The presence of bradycardia may represent an important side effect caused by HEX when compared to other nitric oxide donors and must be further investigated. In addition, we are currently running toxicity tests in order to establish the safety of the HEX. Furthermore, other pharmacological studies to establish the half-life of the compound and the characterization of its potential secondary metabolites are under investigation. Of note, our preliminary data suggest that HEX does not cause tolerance as caused by other nitric oxide donors, which seems to be a promising advantage of this new compound. Regarding its potency compared to other nitric oxide donors, our present and previous studies show that HEX is able to increase nitric oxide with the same magnitude as nitroglycerin (Figure 2) and the 2-nitrate-1,3-dibuthoxypropan (NDBP), a new organic nitrate developed in our laboratory (França-Silva et al., 2012a,b).

Telemetry was used to evaluate blood pressure and heart rate during 7 weeks as well as the HEX effects when administered orally for 7 days when animals were already hypertensive. The treatment was done by adding HEX in drinking water, so that each animal received approximately 10 mg/kg/day, according to the daily water intake. Our work showed that BP increased gradually and significantly over a 5 week period after the clip implantation, reaching a hypertensive level about 6th week in agreement with previous studies (Martinez-Maldonado, 1991; Oliveira-Sales et al., 2014), while the BP in the sham group was not changed over time. Starting at day 42 post renal artery clipping, one hypertensive group was treated with HEX for 7 days and the BP decreased significantly. This result encourages further studies including clinical trials to investigate the potential antihypertensive effect of HEX. However, a better characterization of the side effects, stability, half-life, and toxicity of the compound must be established before we move further.

In conclusion, our data show that HEX is a NO donor, which induces vasorelaxation via NO/cGMP/PKG pathway and activation of the ATP-sensitive K^+^ channel and presents antihypertensive effect in rats with renovascular hypertension.

## Author contributions

Conception and design: LM, VL, MF, VB. Acquisition, analysis and interpretation of data: LM, DDG, DDAG, MF, MB, TD. Analyzed the data: LM, DDG, DDAG, MB, MF, TD, PA, VL, VB. Materials and reagents: PA, VL, VB. Drafting or revising and final approval: LM, DDG, DDAG, MF, TD, PA, VL, VB.

## Funding

This work has been funded by Conselho Nacional de Desenvolvimento Científico e Tecnológico (CNPq), grant numbers 467147/2014-0 (VSL), 472133/2013-6 and 304772/2014-3 (VAB).

### Conflict of interest statement

The authors declare that the research was conducted in the absence of any commercial or financial relationships that could be construed as a potential conflict of interest.
